# Reduce and optimize critical care unit noise

**DOI:** 10.1186/cc14687

**Published:** 2015-09-28

**Authors:** Ana Maria Cavalheiro, Carolina L Pires, Fabiana Aparecida Sarmento, Leonardo José R Ferraz, Neide M Lucino

**Affiliations:** 1 Hospital Albert Einstein, Morumbi, São Paulo, SP, Brazil

## Introduction

Noise is an environmental stressor that is known to have physiological and psychological effects. The body responds to noise in the same way as it responds to stress and over time can impair health. Research clearly shows that hospital noise levels exceed noise level recommendations and have the potential to increase complications in patients. What is less known is the effect Critical Care Unit noise has on nurses.

## Objective

The purpose of this article is to discuss Critical Care Unit noise.

## Methods

By measuring noise during peak and off-peak ICU hours, analysts can detect and correct the noise. Noise meter readings established by a pollution control board offer quantitative readings that help predict, and therefore prevent, future noise problems. We identified the primary sources for noise pollution through a questionnaire applied to these patients and caregivers. Complaints related to undesirable noises were alarms, parametric monitors, and noise caused by the companions of other patients and the multidisciplinary team. Noise measurements were carried out by occupational medicine, which identified the noise peaks as 8:00 p.m. 67 dB, 9:00 a.m. 62 dB and 1:00 p.m. 63.8 dB. The Deming model was adopted and by brainstorming with the multidisciplinary team we decided to use educational strategies, lectures for the multidisciplinary team and bookmark images stimulating silence for companions in the ICU. The lectures to staff were conducted by psychologists and speech therapists about using the voice and care related to behavior and education. We made new measures of noise after 30 days and noticed a significant drop to 45 db in the night period, 54 dB in the morning and 53 db in the afternoon.

## Conclusion

Educational activities within work are still the best way to obtain significant results for healthy behaviors to ensure the safety of patients and professionals.

